# Delta-5-desaturase: A novel therapeutic target for cancer management

**DOI:** 10.1016/j.tranon.2021.101207

**Published:** 2021-08-23

**Authors:** Lizhi Pang, Harshit Shah, Yi Xu, Steven Qian

**Affiliations:** a Department of Pharmaceutical Sciences, North Dakota State University, Sudro 108, 1401 Albrecht Blvd, Fargo, ND, USA; b Department of Cell Systems and Anatomy, UT Health San Antonio, San Antonio, TX, USA

**Keywords:** Delta-5 desaturase, Prostaglandin E_2_, 8-hydroxyoctanoic acid, Tumor inflammation, Polyunsaturated fatty acid metabolism, PUFAs, polyunsaturated fatty acids, D5D, delta-5-desaturase, DGLA, dihomo-γ-linolenic acid, COX-2, cyclooxygenase-2, PGs, prostaglandins, PGE_2_, prostaglandin E_2_, AA, arachidonic acid, ETA, eicosatetraenoic acid, EPA, eicosapentaenoic acid, EDA, eicosadienoic acid, SNP, single-nucleotide acid polymorphism, ER, endoplasmic reticulum, 8-HOA, 8-hydroxyoctanoic acid, RNAi, RNA interference, HDAC, histone deacetylases, TCGA, the cancer genome atlas, LTs, leukotrienes, LXR, liver X receptor, PPARα, peroxisome proliferator-activated receptor α, RXR, retinoid X receptor, SREBP-1c, sterol regulatory element-binding protein 1c, 5-LOX, 5-lipoxygenase, EMT, epithelial-mesenchymal transition, MDSCs, myeloid-derived suppressor cells, DNMT3B, DNA methyltransferase 3B, ROS, reactive oxygen species, YAP1, yes-associated protein-1, TAZ, transcriptional coactivator with PDZ-binding motif, TME, tumor microenvironment, Bax, Bcl-2-associated X protein, MMP, matrix metalloproteinase, GPX4, glutathione peroxidase 4, ELOVL5, elongation of very long-chain fatty acid protein 5, POBN, α-(4-Pyridyl 1-oxide)-N-tert-butylnitrone, 5-FU, fluorouracil, EpCAM, epithelial cell adhesion molecule, 3WJ, 3-way junction, HAT, histone acetyltransferase

## Abstract

Delta-5 desaturase (D5D) is a rate-limiting enzyme that introduces double-bonds to the delta-5 position of the n-3 and n-6 polyunsaturated fatty acid chain. Since fatty acid metabolism is a vital factor in cancer development, several recent studies have revealed that D5D activity and expression could be an independent prognostic factor in cancers. However, the mechanistic basis of D5D in cancer progression is still controversial. The classical concept believes that D5D could aggravate cancer progression via mediating arachidonic acid (AA)/prostaglandin E_2_ production from dihomo-γ-linolenic acid (DGLA), resulting in activation of EP receptors, inflammatory pathways, and immunosuppression. On the contrary, D5D may prevent cancer progression through activating ferroptosis, which is iron-dependent cell death. Suppression of D5D by RNA interference and small-molecule inhibitor has been identified as a promising anti-cancer strategy. Inhibition of D5D could shift DGLA peroxidation pattern from generating AA to a distinct anti-cancer free radical byproduct, 8-hydroxyoctanoic acid, resulting in activation of apoptosis pathway and simultaneously suppression of cancer cell survival, proliferation, migration, and invasion. Hence, understanding the molecular mechanisms of D5D on cancer may therefore facilitate the development of novel therapeutical applications. Given that D5D may serve as a promising target in cancer, in this review, we provide an updated summary of current knowledge on the role of D5D in cancer development and potentially useful therapeutic strategies.

## Highlights

• D5D is an independent prognostic factor in cancer.
• D5D aggravates cancer progression via mediating AA/PGE_2_ production from DGLA.
• AA/PGE_2_ promotes cancer progression via regulating the tumor microenvironment.
• Inhibition of D5D redirects COX-2 catalyzed DGLA peroxidation, producing 8-HOA.
• 8-HOA suppress cancer by regulating proliferation, apoptosis, and metastasis.

## Graphical abstract

**Figure:**
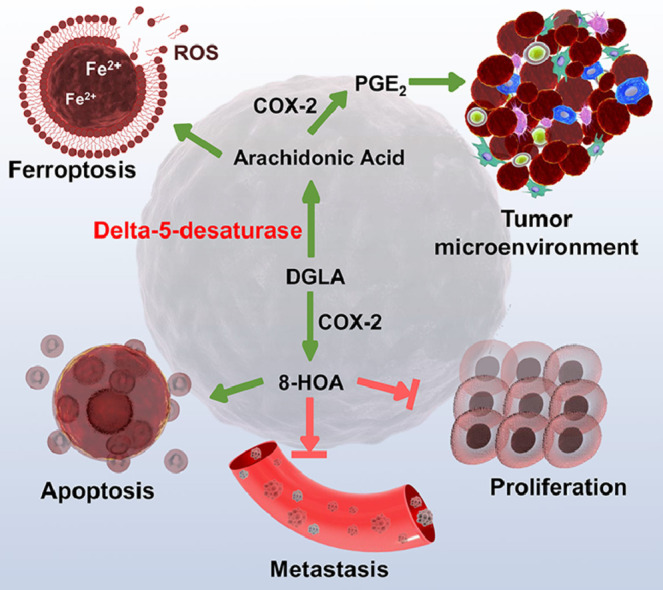

## Introduction

Delta-5 desaturase (D5D) is a rate-limiting enzyme in polyunsaturated fatty acids (PUFAs) synthesis for introducing double-bonds to the delta-5 position in the fatty acid chain [1]. Given the key role of the PUFAs synthesis pathway in energy homeostasis, D5D has been widely studied in metabolic diseases, such as hepatic steatosis and type 2 diabetes [2,3]. The two main functions of D5D are (1) to catalyze eicosatetraenoic acid (ETA) to eicosapentaenoic acid (EPA) in the n-3 PUFAs pathway, and (2) to catalyze dihomo-γ-linolenic acid (DGLA) to arachidonic acid (AA) in the n-6 PUFAs pathway [1]. Although the effect of the n-3 PUFAs pathway on cancer has been widely studied, the function of the n-6 PUFAs pathway, especially D5D, on cancer has remained poorly understood. However, recent studies have revealed that D5D may also play a crucial role in cancer by regulating inflammation, ferroptosis, apoptosis, proliferation, and metastasis [4], [5], [6], [7]. Therefore, it is critical to find out how D5D participates in diverse mechanisms so researchers can develop new therapeutical strategies with the proper fatty acids supplementation and fulfill the promise of precision medicine.

## The structure, distribution, and function of D5D

D5D is coded by the *FADS1* gene, which is located on chromosome 11q12-13.1 [8]. *FADS1* and other subtypes of *FADS* genes consist of 11 introns and 12 exons and occupy 100 kb regions in total. The variants of the *FADS* gene cluster have been reported to be closely associated with fatty acid consumption, pregnancy, auto-immune diseases, and cancers. D5D is a typical front-end fatty acyl-CoA desaturase, which binds to the endoplasmic reticulum (ER) membrane and catalyzes the synthesis of downstream PUFAs. The D5D enzyme belongs to the superfamily of iron-dependent enzymes that consists of two domains, cytochrome b5 domain at N-terminal with heme-binding motifs and desaturase domain at C-terminal with histidine boxes where one of the histidine residuals is replaced by glutamine, which is responsible for the activity of desaturase. The replacement of the glutamine back to histidine (or isoleucine) could result in abrogating the desaturase's activity [1,9]. However, the precise crystal structure of D5D is still unclear as it is difficult to stabilize the membrane-bound enzyme with full activities [10]. Since the D5D and D6D shared many similarities in gene SNP, protein structure, and function, it could be a challenge to isolate enzymes exclusively exhibiting D5D activity on their substrates (DGLA and ETA) rather than catalyzing on broad PUFAs’ precursors.

Fatty acid desaturases are widely distributed in most animal tissues, and are essential to synthesize PUFAs for energy supply and signal transduction (serve as first/second messengers). The D5D enzyme is highly expressed in the brain, lung, pancreas, liver (highest), and variety of cancer cells [1]. The low D5D activity in serum lipids has been identified as a potential biomarker in the prediction of disease status, such as metabolic risk in children, hepatic steatosis, type 2 diabetes, and cancers [3,[11], [12], [13]]. A recent The Cancer Genome Atlas (TCGA) database analysis has indicated that the mRNA expression levels of *FADS1* are increased in head-neck squamous cell carcinoma tissues [14].

**Fig. 1:**
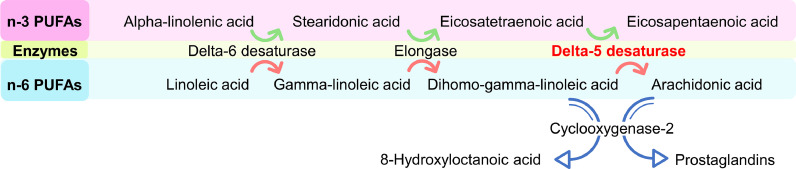
*Pathway of n-3 and n-6 polyunsaturated fatty acids (PUFAs) synthesis.* The n-3 PUFAs, such as Alpha-linolenic acid is metabolized by delta-6-desaturase and elongase to Eicosatetraenoic acid, which further peroxidized by Delta-5-Desaturase to Eicosapentaenoic acid (Shown in red row). The same sets of enzymes metabolize n-6 PUFAs, linoleic acid, to Arachidonic acid (n-6 metabolism shown in blue). The COX-2 is responsible for conversion of arachidonic acid to precancerous prostaglandins, whereas COX-2 is also involved in peroxidation of Dihomo-gamma-linoleic acid to an anti-cancer compound, 8-HOA (For interpretation of the references to color in this figure legend, the reader is referred to the web version of this article.).

D5D in the n-3/-6 PUFAs pathway is responsible to convert dietary essential fatty acids (α-linolenic acid and linolenic acid) to a series of downstream PUFAs by introducing double-bonds to the fatty acid chain from the carboxylic end, increasing unsaturation (Fig. 1). Another type of desaturase catalyzing PUFAs synthesis from methyl-end to pre-existing double bonds, such as D12D and D15D, are not present in humans/mammals. Therefore, mammals have to uptake α-linolenic acid and linoleic acid from their daily diet to maintain the balance of fatty acid compositions. Saturated fatty acids have a higher transition temperature than unsaturated fatty acids. Therefore, the ratio of saturated to unsaturated fatty acid is considered as the primary determinant of the melting temperature of triglycerides for regulating cellular membrane fluidity [1,10,15]. In our daily diet, α-linolenic acid and linoleic acid are the major resource of n-3 and n-6 fatty acids, respectively [16]. Both n-6/-3 fatty acid synthesis pathways share the same set of enzymes, including D6D, elongase, and D5D, of which D5D is in response to catalyze the formation of EPA in the n-3 pathway and AA in the n-6 pathway [8].

## D5D implication in PUFA related diseases including cancer

EPA and AA are the precursors of a variety of inflammatory mediators, such as prostaglandins (PGs) and leukotrienes (LTs), for regulating physiological and pathological functions [17]. Selective knocking down *FADS1* by *in vivo* antisense oligonucleotide could induce hepatic inflammation and atherosclerosis in mice [18,19]. *In vitro* study have further confirmed that the knockdown of *FADS1* activates classic M1 macrophages and inhibits M2 activation, and causes alterations of liver X receptor (LXR) associated gene expression, implicating the key role of D5D in liver lipid metabolism [19]. Additionally, a population-based Kuopio Ischaemic Heart Disease Risk Factor Study has suggested that higher D5D activity is favorably associated with stroke and metabolic risk factors, including low systolic/diastolic blood pressure, insulin level, C-reactive protein concentrations, BMI, and better homeostatic model assessment indices [20]. Additionally, high DGLA concentration and low D5D activity are valuable predictors of hepatic steatosis [2]. D5D also has been identified as a risk factor of type 2 diabetes. The level of plasma apoB is negatively correlated with D5D activity [3]. Moreover, the D5D activity is also negatively associated with serum triiodothyronine in adolescents with eating disorders, indicating that D5D may participate in thyroid hormone regulation [21].

Several rodent studies have reported that the expression and activity of D5D could be influenced by diet. Indeed, the PUFAs synthesis is regulated under a strong feedback pathway [1]. The D5D is inhibited by dietary PUFAs, especially the high-fat diet [22]. Different transcription factors, including peroxisome proliferator-activated receptor α (PPARα), retinoid X receptor (RXR), sterol regulatory element-binding protein 1c (SREBP-1c), have been reported with a strong regulator effect on D5D expression [1]. All three desaturases, D5D, D6D, and D9D, can be activated by SREBP-1c via regulating mRNA expression. It has been observed that mice with overexpression of SREBP-1a, -1c, and -2 have higher expression of hepatic D5D than the wild-type mouse. After a fast/fed cycle, supplementation of a carbohydrate diet with linoleate or EPA significantly inhibits D5D expression, indicating that PUFAs could trigger the feedback suppression of D5D [23]. Another transcription factor PPARα controls β-oxidation in the liver [1]. Activation of PPARα could induce D5D expression in fast conditions. The cross-talk between SREBP-1c and PPARα may build the basic mechanism of how D5D is regulated under different nutritional statuses and energy states [18,23]. A recent study based on UK Biobank has revealed the positive association between FADS1 with rs174561 variant in the frontal cortex and daytime napping. Moreover, two-sample Mendelian randomization analyses have confirmed that more frequent daytime napping is correlating to independent risk factors (waist circumference and blood pressure) of cardiometabolic diseases [24]. Given the fact that many downstream inflammatory mediators are generated by the D5D-catalyzed n-6 PUFAs pathway, the correlation between daytime napping and cardiometabolic risks may be explained by activation of AA/PGs-mediated inflammatory response. Of note, the data of this study has been collected from individuals living in the United Kingdom where the Western diet is the mainstream. However, the D5D expression and activity may vary in patients with different diets and PUFAs uptake. For example, an iron-rich diet could decrease D5D activity but increase D5D mRNA expression in male Wistar rats [25]. A comparative study compares the D5D gene expression between Europeans and Chinese. It has been found that Europeans with high saturated fatty acid and less PUFAs’ diet express higher D5D genes in peripheral blood mononuclear cells, indicating the strong correlation between dietary fatty acid uptake and D5D activity [22]. Therefore, the relationship between daytime napping, D5D expression/activity, and diet may need to be investigated in more populations with different dietary preferences.

More studies recently have revealed the potential correlation between D5D expression and cancer [13,26,27]. Given the key role of inflammation in cancer, the D5D may be involved in carcinogenesis via mediating PUFAs metabolism in the tumor microenvironment. However, the correlation between D5D and cancer development is still controversial (Table 1). Studies have suggested that a low D5D level is a predictor of worse prognosis in non-small cell lung cancer and esophageal squamous cell carcinoma. Patients with low *FADS1* expression appear poor overall survival and disease-free survival [27,28]. On the contrary, other studies have demonstrated that high expression of D5D is an indicator of cancer progression. A recent TCGA based study has suggested that bladder cancer patients with high *FADS1* expression have a poor prognosis [26]. The *in vitro* study further shows that overexpression of *FADS1* could lead to bladder cancer proliferation, whereas the *FADS1* knockdown could arrest the cell cycle [13]. Moreover, *FADS1* could be upregulated by a long non-coding RNA linc00460 in osteosarcoma, resulting in distant metastasis and reduced overall survival [29]. A genome-wide association study (GWAS) of colorectal cancer in East Asians has demonstrated that *FADS1* expression in colon tumor tissues is higher than normal tissues [30]. Additionally, *FADS1* is also upregulated in patients with hepatocellular carcinoma [31]. Why the opposite conclusions have been made in the above-listed studies? One possible explanation is that the function of D5D may vary in different organs and cancers. Although D5D is a critical enzyme for the synthesis of PUFAs, other enzymes, such as D6D, cyclooxygenase (COX), 5-lipoxygenase (5-LOX), need to be coupled with D5D to generate corresponding eicosanoids [17]. The function of D5D in cancer may be influenced by the status of other enzymes [32]. Diet and uptake of PUFAs could be another reason. For instance, the negative correlations of *FADS1* with lung cancer and esophageal squamous cell carcinoma have been established by analyzing tumor samples from patients in the same region [27,28]. Given the fact that D5D expression and activity could be regulated by diet, the interrelationships and relevance of D5D to cancer development may vary in patients from different regions with disparate food and PUFAs preferences. Therefore, more studies may need to be conducted to further validating the function of D5D in cancer in larger populations across different regions. Moreover, not only *FADS1* expression, but also certain SNPs of *FADS1*, such as rs174549, rs174548, and rs174550 have been identified as independent and favorable factors in predicting oral, lung, colorectal cancers, and laryngeal squamous cell carcinoma progression [33], [34], [35], [36].

**Table 1:** Potential prognostic and/or predictive utility of D5D and its partners in cancers.

Molecules	Cancer type	Clinical outcomes	Prognostic/ predictive utility	Ref.
D5D	Non-small-cell lung cancer	(1) Higher expression in normal bronchial mucosa than tumor tissues (2) Negatively associates with tumor size and histological grade (3) Lower expression associates with shorter overall survival and disease-free survival	Protective	[27]
D5D	Esophageal squamous cell carcinoma	Associates with better disease-free survival and overall survival	Protective	[28]
D5D	Bladder cancer	(1) Positively associates with tumor grade (2) Enhances the proliferation	Poor prognosis	[13]
D5D	Laryngeal squamous cell carcinoma	High expression and bioactivity in tumor tissues	Undetermined	[14]
D5D rs174549	Oral cancer	Correlates to a decreased risk of oral cancer	Protective	[37]
D5D rs174548	Lung cancer	(1) Smoking-independent (2) Particularly associates with lung cancer in women	Undetermined	[34]
COX-2	Hypopharyngeal carcinoma	Associates with chemoresistant	Undetermined	[38]
COX-2	Colorectal cancer	Relates to susceptibility to cancer in Caucasians	Increased risk	[39]
COX-2	Osteosarcoma	(1) Higher expression in osteosarcoma than benign osteochondroma (2) Associates with tumor grade, clinical stage, and metastasis	Poor prognosis	[40]
COX-2	Breast cancer	High expression associates with poor overall survival	Poor prognosis	[41]
COX-2	Head and neck cancer	(1) Highly associates with a high risk of lymph node metastasis and advanced TNM stage (2) Poor survival effect	Poor prognosis	[42]
Linc00460	Osteosarcoma	Positively correlates with distant metastasis and poor overall survival	Poor prognosis	[29]
AA	Colorectal cancer	Positively associates with colorectal cancer	Increased risk	[43]
PGE_2_	Gastric cancer	PGE-M (a urinary metabolite of PGE_2_) is associated with a higher risk of gastric cancer	Increased risk	[44]

## D5D regulates cancer progression via mediating prostaglandin E_2_ (PGE_2_) production

**Fig. 2:**
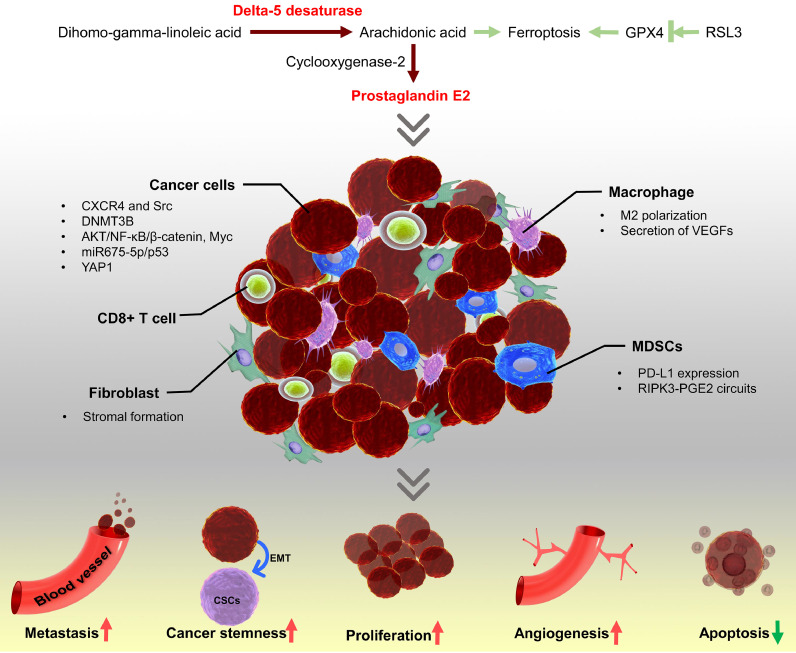
Delta-5 desaturase (D5D) regulates cancer progression via mediating arachidonic acid (AA) and prostaglandin E2 (PGE_2_) production. DGLA/AA/PGE_2_ axis promotes cancer progression via increasing metastasis, cancer stemness, proliferation, angiogenesis, and decreasing apoptosis as shown in red and green arrows (bottom panel in the figure). The light green arrows indicate the role of DGLA and AA in ferroptosis (upper panel in the figure) (For interpretation of the references to color in this figure legend, the reader is referred to the web version of this article.).

The role of fatty acid metabolism in cancer has been extensively studied as fatty acids could serve as an alternative fuel for providing energy to support cancer cell growth and proliferation [45]. However, fatty acids also contribute to tumorigenesis by mediating various signaling pathways. D5D is a rate-limiting enzyme for catalyzing the formation of AA/EPA from DGLA/ETA [8]. The newly formed AA could be continuously transformed to PGE_2_ by COX-2 and PGE synthase in cancer cells [17]. As the most abundant prostaglandin, PGE_2_ is involved in many aspects of tumorigenesis [46]. COX-2/PGE_2_ axis contributes to the formation of the inflammatory microenvironment in the tumor tissues, resulting in proliferation, invasion, epithelial-mesenchymal transition (EMT), cancer cell stemness, and inhibition of apoptosis via regulating the function of macrophages, cancer-associated fibroblasts, CD8^+^ T cells, and myeloid-derived suppressor cells (MDSCs) (Fig. 2) [39], [40], [41], [42], [43], [44], [45], [46], [48], [49], [50], [51]. Given the vital role of inflammation in cancer, the over-activation of the COX-2/PGE_2_ pathway could upregulate the protein expression of phospho- NF-κB p65, resulting in invasion and proliferation in ovarian cancer cells [54]. In breast cancer cells, DNA methyltransferase 3B (DNMT3B) can be induced by PGE_2_, leading to a significant epigenetic change in tumors and metastatic sites [55]. Tumor growth and migration are also depending on angiogenesis for transporting nutrients and chemical signals. The activation of PGE_2_-EP2/4 could promote angiogenesis through CXCR4 and upregulation of Src [56,57]. Additionally, Orai1 has recently been identified as one of the downstream molecules of the EP4/PI3K pathway, which is responding to PGE_2_-induced cancer cell migration [58]. The increased PGE_2_ in cancer cells could further trigger a positive activation loop of YAP1/COX-2/EP4, resulting in colorectal cancer cell proliferation and polyp formation [59]. Not only inhibition of the immune system, but also a recent study has revealed that PGE_2_ could promote colorectal progression by upregulation of miRNA 675-5p (miR675-5p) [60]. Additionally, other miRNAs and molecules, such as miR-370-3p, miR-206, miR-146a, syntenin-1 also could regulate PGE_2_ production and inactivation in colorectal cancer [61], [62], [63].

Despite the importance of PGE_2_ synthesis in cancer, the interrelationships and relevance of D5D expression and activity in PGE_2_-mediated cancer progression remain poorly understood. One reason is that compared to D5D, COX-2 is a more conspicuous and accessible target for regulating PGE_2_ production, because the structure and function of COX-2 have been widely studied. Many studies have demonstrated the effect and mechanism of COX-2 inhibitors on various types of cancers *in vitro* and *in vivo*. However, long-term and high-dose treatment of COX-2 inhibitor is required to boost the PGE_2_ inhibition in clinical practices [64,65]. Worth noting, not only in cancer cells but also COX-2 is the key molecule regulating inflammation and other physiological function throughout the body. Thus, the usage of COX-2 inhibitors in cancer treatment may raise safety concerns. Indeed, a significant increase in cardiovascular side effects has been observed in the clinical study when cancer patients received the addition of COX-2 inhibitors in the standard treatment [66]. Moreover, another study has suggested that COX-2 inhibitors fail to improve the overall and progression-free survival in cancer patients irrespective of COX-2 expression in tumors [67]. Therefore, continued research into upstream molecules of COX-2/PGE_2_ pathways, such as D5D could help us to better understand the role of fatty acid metabolism in cancer progression and to overcome the limitation of COX-2 inhibitors.

## D5D regulates cancer progression through activating ferroptosis

Ferroptosis is the iron-dependent mechanism of cell death and is affected by cellular redox status [68,69]. Although ferroptosis shared many biochemically and morphologically features with necrosis, such as chromatin condensation, cytoplasmic swelling, and damage of plasma membrane integrity, investigators believe that ferroptosis is a distinct cell death pathway differing from necrosis, apoptosis, and autophagy [69], [70], [71], [72]. Compare to other cell death mechanisms, ferroptosis is featured by lipid peroxidation and iron accumulation. It has been found that iron metabolism, especially cellular iron versus systemic iron level, plays a key role in ferroptosis [68]. Iron could promote ferroptosis by inducing reactive oxygen species (ROS) generation via Fenton reaction or increasing the activity of prolyl hydroxylase and lipoxygenase [68]. Additionally, products derived from PUFAs peroxidation, such as hydroperoxides and 4-hydroxynonenal may also lead to ferroptotic cell death [73]. Among all the non-/oxygenated phospholipid species, doubly and triply oxygenated AA and adrenic acid-containing phosphatidylethanolamine species are the most important phospholipids in the ferroptosis pathway [74]. The generation of these derivates during ferroptosis requires the function of two genes, *ACSL4* and *LPCAT3* [70,[74], [75], [76]]. Notably, a recent study has suggested that DGLA (precursor of AA) may lead to ferroptosis in cancer cells [77]. It has been observed that the DGLA-induced cancer cell death could be blocked by Ferrostatin-1, which is a selective inhibitor of ferroptosis, indicating that DGLA is a ferroptosis inducer [77]. Since D5D is the primary enzyme for AA production from DGLA, it is plausible that D5D plays a key role in ferroptosis. D5D expression and activity may affect the ferroptosis pathway via regulating the content of n-3/n-6 PUFAs in cancer cells.

Studies have demonstrated that induction of ferroptosis could inhibit cancer development and enhance the efficacy of chemotherapy, targeted therapy, and radiotherapy [78]. The role of D5D and lipid peroxidation in ferroptosis has been recently explored in cancer cells. The differential expression of D5D has been observed between intestinal-type and mesenchymal-type gastric cancer cells. By knocking out the D5D gene or using small molecule D5D inhibitor CP-24879, researchers have demonstrated that the expression and activity of D5D are positively correlated with the ferroptosis sensitivity of gastric cancer cells. Indeed, inhibition of D5D diminishes the glutathione peroxidase 4 (GPX4) inhibitor RSL3-induced ferroptosis in YCC-16 gastric cancer cells. However, the addition of AA to D5D depleted gastric cancer cells could reverse the effect of D5D inhibition, restoring the ferroptosis sensitivity of cancer cells (Fig.2). Not only D5D but also elongation of very long-chain fatty acid protein 5 (ELOVL5) is essential for ferroptosis. During n-6 PUFA synthesis, ELOVL5 catalyzes gamma-linolenic acid to DGLA, which is the substrate of D5D. Inhibition of ELOVL5 decreases the sensitivity of cancer cells to ferroptosis [5]. However, the addition of exogenous D5D substrate (DGLA) triggers cell death via activating the ferroptosis pathway in fibrosarcoma cells. Worth noting, this phenomenon has been observed in cells treated with a high dose of DGLA (500 µM), whereas low dose (less than 250 µM) appears no anti-cancer effect *in vitro* [77]. Therefore, D5D may serve as a central checkpoint in ferroptosis via manipulating PUFA synthesis [5].

## Inhibition of D5D as a new strategy for cancer treatment

Since D5D is an independent prognostic biomarker in many types of cancers, targeting D5D seems like a promising alternative strategy for cancer therapy. By transfecting laryngeal cancer cells with lentivirus vector with *FADS1* shRNA, Zhao et al. have demonstrated that D5D knockdown could inhibit cancer cell proliferation and migration. Additionally, D5D knocking down cells also exhibit the prolonged G1 phase and more apoptosis, indicating that inhibition of D5D expression could suppress laryngeal cancer cell growth. The microarray assay and protein-protein interaction network further indicate that the effect of D5D knocking down may associate with the AKT/mTOR pathway. Studies have demonstrated that PGE_2_ could activate PI3K/AKT/mTOR pathway via acting through EP2/4 receptors, increasing AKT, p670S6K, and S6 phosphorylation in cancer cells [79]. Since silencing D5D decreases PGE_2_ production, it has been found that AKT/mTOR pathway is inactivated in tumor tissues after D5D knockdown [14]. Although more studies need to be done to elucidate how PGE_2_ connects D5D and AKT/mTOR pathway, it is plausible that the effect of D5D on the AKT/mTOR pathway may depend on PGE_2_ production in cancer cells [14].

**Fig. 3:**
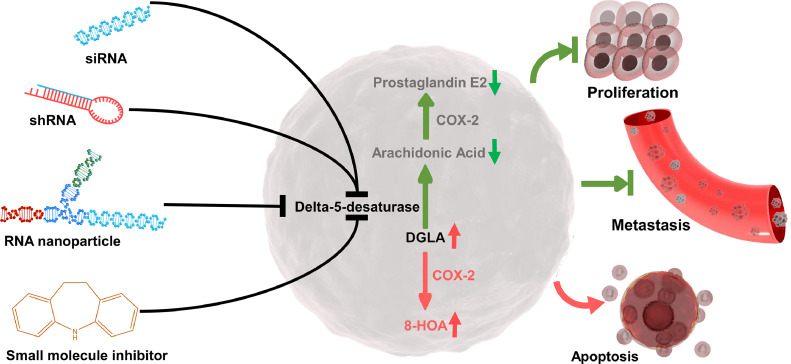
Inhibition of D5D as a new strategy for cancer treatment. Inhibition of D5D suppresses the AA/PGE_2_ generation in cancer cells as shown in green arrows. The alternative pathway could be activated to promote 8-HOA formation from COX-2 catalyzed DGLA peroxidation as shown in red arrows (For interpretation of the references to color in this figure legend, the reader is referred to the web version of this article.).

Not only inhibition of PGE_2_ generation, but suppression of D5D may also boost free radical reactions by reprogramming COX-2-catalyzed DGLA peroxidation (Fig. 3). Xiao et al. have identified a series of free radicals from DGLA peroxidation by LC/ESR/MS with α-(4-Pyridyl 1-oxide)-N-tert-butylnitrone (POBN), which is a spin trap for stabilizing free radicals. Two unique free radicals (POBN adducts) have been identified from DGLA/COX-2 C-8 oxygenation, POBN/ ^•^C_7_H_13_O_2_ (m/z 324) and POBN/ ^•^C_8_H_15_O_3_ (m/z 354). These two free radicals appear exclusively in DGLA peroxidation, but not AA peroxidation, indicating that alternative downstream pathways of DGLA are existing rather than the AA/PGE_2_ pathway [80]. Gu et al. have further improved the spin trapping/solid-phase extraction approach and investigated the association between these two free radicals and colon cancer cell growth. In a cellular environment, ^•^C_7_H_13_O_2_ and ^•^C_8_H_15_O_3_ could immediately capture hydrogen to form corresponding derivates, heptanoic acid, and 8-hydroxyoncanoic acid (8-HOA). MTS assay and cell cycle analysis suggest that 8-HOA could decrease the cell viability and delay the G_1_ phase of HCA-7 colon cancer cells. However, heptanoic acid does not affect the cancer cell cycle and proliferation [81]. To better understand the role of DGLA-derived free radicals in cancer, Xu et al. have assessed the apoptosis of HCA-7 colon cancer cells treated with 8-HOA or hexanol. 8-HOA (1 μM) significantly induces apoptosis in colon cancer cells by regulating the protein expression of p53 and procaspase-9. However, the effect of other DGLA's free radical derivates (hexanol and heptanoic acid) on apoptosis is modest. Moreover, 8-HOA also drops the IC50 of fluorouracil (5-FU) on HCA-7 cells from 1 mM to 0.5 mM, indicating that 8-HOA may serve as the supplementary treatment for chemotherapy [82]. Moreover, Yang et al. have found that 8-HOA also could suppress the BxPC-3 pancreatic cancer cell proliferation and promote apoptosis. Additionally, the wound healing assay suggests that 8-HOA could inhibit the migration of HCA-7 and BxPC-3 cancer cells. The addition of 8-HOA significantly enhances the efficacy of gemcitabine (first-line chemo) on pancreatic cancer. Interestingly, the increased protein expression of acetyl-histone H3 has been observed in both colon and pancreatic cancer cells treated with 8-HOA, indicating that histone deacetylase (HDAC) may act as the downstream effector of 8-HOA [83]. The HDAC activity response curve to 8-HOA has been established on A549 lung cancer cells [6]. A similar effect of 8-HOA also has been observed in breast cancer cells (MDA-MB 231 and 4T1) [7]. Therefore, it is reasonable to believe that 8-HOA is a broad-spectrum antitumor agent that regulating many aspects of cancer progression.

However, it has been observed in the above-mentioned studies that the treatment of 8-HOA alone without combination with other chemotherapies only can result in less than 30% of inhibition of cancer cell growth. One possible reason is that the effect of exogenous 8-HOA cannot fully represent the effect of endogenous 8-HOA, which is derived from ^•^C_8_H_15_O_3_ [80]. Although the free radical form of 8-HOA (^•^C_8_H_15_O_3_) is transient in the cellular environment, it may still provide additional cytotoxicity rather than the derivate form of 8-HOA (C_8_H_16_O_3_). However, in normal cellular conditions, the free radical form of 8-HOA (^•^C_8_H_15_O_3_) is not the main product of DGLA, which is more likely to be converted to AA by D5D. To enforce the generation of 8-HOA, Xu et al. have knocked down D5D in HCA-7 cells by siRNA transfection. The DGLA consumption is significantly slower in D5D knockdown cells compared to cells with full D5D expression. After 48 h of the transfection, the 8-HOA level increases from ∼0.3 μM to more than 0.8 μM, indicating the activation of the DGLA/8-HOA pathway. It has been observed that supplementation of DGLA (100 μM) to D5D knockdown cells could activate p53-dependent apoptosis and enhance the effect of 5-FU, irinotecan, and regorafenib [84]. A similar effect of D5D siRNA-based treatment has also been found in pancreatic and breast cancer cells [85,86]. By cross-comparison of all these studies, breast cancer cells (4T1 and MDA-MB 231) generate more 8-HOA (∼1.2 μM) than colon and pancreatic cancer cells (∼0.8 μM). Interestingly, an *in vitro* threshold of endogenous 8-HOA level (0.5 μM) has been identified in these studies. The effect of D5D knockdown only can be elicited on cancer cells if the threshold (0.5 μM 8-HOA) is achieved [84], [85], [86]. Therefore, the effectiveness of the long-term exposure of just above the threshold concentration 8-HOA may be superior to a high concentration 8-HOA with a short exposure time on cancer cells. This phenomenon may also explain why a high concentration of exogenous 8-HOA appears a lower efficacy than a low concentration of DGLA-derived 8-HOA. The endogenous 8-HOA could be continuously produced from DGLA, resulting in above the threshold concentration of 8-HOA for a long period in cancer cells.

Despite the high efficiency of D5D siRNA, the off-target effect and stability are still the major concerns of RNA-based drugs, especially in animal models and patients [87]. To ensure the continuous generation of 8-HOA, Yang et al. have established the stable D5D knockdown cancer cells using shRNA transfection. Supplementation of DGLA to D5D shRNA transfected BxPC-3 and HCA-7 cancer cells inhibit the protein expression of matrix metallopeptidase 2 and 9 (MMP2/9), which are key enzymes for degradation of type IV collagen, extracellular matrix, and basement membrane [83]. Additionally, knocking down D5D triggers the expression of cell adhesion protein E-cadherin but decreases the expression of structural protein vimentin and snail, which are critical proteins for EMT during cancer metastasis [7]. Not only exogenous 8-HOA, but DGLA-derived endogenous 8-HOA also enhances the inhibitory effect of gemcitabine and 5-FU on pancreatic and colon cancer cells possibly through co-regulating transcriptional activation of genes in cancer metastasis pathways [83]. Furthermore, the *in vivo* study has demonstrated that D5D shRNA transfected tumor tissues only have about a half expression of D5D compared to mice bearing wild-type tumors. Lacking D5D expression limits the AA generation and significantly improves the 8-HOA production from ∼0.1 to ∼0.5 µg/g in tumor tissues. Consequently, about 26% tumor size reduction has been observed in nude mice with D5D shRNA transfection [88].

To further improve the efficiency of the D5D knocking down approach, Xu et al. have employed the targeted 3-way junction (3WJ) RNA nanoparticle to deliver the D5D siRNA to the tumor region [89]. Unlike other nanoparticles, RNA nanoparticle does not encapsulate the drug/siRNA in the center; however, all the therapeutical siRNA/miRNA are directly conjugated on the branches of the RNA nanoparticle core [90]. The excellent thermodynamical and chemical stability of the 3WJ RNA nanoparticle improves the *in vivo* performance of D5D siRNA. Moreover, tumor-targeting is achieved by adding epithelial cell adhesion molecule (EpCAM) aptamer to one strand of the 3WJ RNA nanoparticle [89]. EpCAM specifically expresses in various types of cancer cells, including colon, lung, and breast cancer cells [91]. Thus, EpCAM aptamer could ensure the binding between nanoparticles and cancer cells. In the xenograft colon cancer model, EpCAM-D5D siRNA nanoparticle significantly increases the 8-HOA production, resulting in about 70% tumor reduction [89]. Shah et al. further expand the benefit of EpCAM-D5D siRNA nanoparticle to breast cancer therapy. Supplementation of DGLA along with EpCAM-D5D siRNA nanoparticle suppresses the tumor growth in orthotopic breast cancer model *via* activating the intrinsic apoptotic pathway. Moreover, they have confirmed that EpCAM-D5D siRNA nanoparticles could inhibit the metastasis of breast cancer cells from the fourth mammary pad to the lung [7]. However, the safety and targeting efficiency of this EpCAM-D5D siRNA nanoparticle on tumor treatment is still unknown. Pang et al. have explored the targeting efficiency and safety of EpCAM-D5D siRNA nanoparticles in lung cancer by comparing the internalization and effect of this nanoparticle on cancer cells versus normal cells. More nanoparticle internalization has been found in A549 lung cancer cells than BEAS-2B normal lung epithelial cells after 4 h of incubation. The selectivity of this nanoparticle is contributed by the EpCAM aptamer that allows nanoparticles to specifically bind to lung cancer cells with high EpCAM expression, whereas it avoids harassing normal cells with low EpCAM expression. Furthermore, it has been observed that the effect of EpCAM-D5D siRNA nanoparticles on A549 cells is better than H1299 lung cancer cells, which have less COX-2 expression. Thus, not only EpCAM but also COX-2 overexpression seems an essential premise for eliciting the effect of EpCAM-D5D siRNA nanoparticles on cancer [6]. Given the fact that COX-2 is the main enzyme for regulating inflammation, more studies need to be conducted to investigate the role of D5D and D5D inhibition-based strategies in the inflammatory tumor microenvironment by involving cytokines and immune cells in consideration. More importantly, since anti-inflammatory agents are widely used in cancer patients for co-treatment of cancer-associated symptoms, patients may gain extra benefit from the possible synergy effect between D5D inhibition-based strategy and anti-inflammatory treatments.

Previous studies have demonstrated that D5D could regulate many aspects of cancer progression, including survival, proliferation, migration, invasion, apoptosis, and cell cycle arrest [6,7,[84], [85], [86]]. It raises the question of how this D5D inhibition-based strategy results in such diverse effects on cancer. One possible explanation is that D5D inhibition could break the bridge between the PGE_2_ and PI3K/Akt/mTOR pathway, which is participated in almost all the aspects of cancer progression [14]. For instance, the PI3K/Akt pathway can activate mTOR, resulting in cancer cell growth and proliferation; Akt regulates BAX/Bcl-2 balance, caspase cleavage, and PARP activation, suppressing apoptosis [92]. Furthermore, given that ULK, FIP200, and ATG13 can be suppressed by mTOR, inhibition of D5D may trigger autophagy in cancer cells [93]. The diverse anti-cancer properties of D5D inhibition may also be attributed to the DGLA-derived 8-HOA, which shared a similar structure with the classical HDAC inhibitor valproic acid. The previous studies have indicated that the overall activity of HDAC in cancer cells can be inhibited by 8-HOA without influencing histone acetyltransferase (HAT) activity. Additionally, the activity of sirtuins (class III HDACs, NAD^+^ dependent) is consistent in cancer cells before and after D5D siRNA transfection. Therefore, 8-HOA may inhibit cancer cell growth via inhibiting HDAC I, II, and/or IV (Zinc dependent) [6]. Moreover, 8-HOA also could regulate YAP1/TAZ pathway and downstream molecules in cancer cells. Indeed, it has been observed in lung cancer cells that supplementation of DGLA and treatment of EpCAM-D5D siRNA nanoparticle suppresses the protein expression and nuclear translocation of YAP1 and TAZ [6]. As the downstream effector of the Hippo pathway, YAP1/TAZ can be activated when Hippo is off, resulting in the promotion of cancer cell growth and proliferation. Interestingly, this phenomenon has not been observed in cancer cells treated with DGLA or EpCAM-D5D siRNA nanoparticles alone, implicating the crucial role of DGLA-derived 8-HOA in YAP1/TAZ signaling [6]. Not only genetically knocking down, but small molecule D5D inhibitor may also hold promise for cancer treatment. However, classical D5D inhibitors, such as sesamin, curcumin, and CP-24879 could not specifically block the activity of D5D [94], [95], [96]. Thus, even though these inhibitors display an ideal inhibitory effect on cancer, it is unconvincing that the effect of these inhibitors is exclusively coming from D5D inhibition. For instance, CP-24879 is a mixed D5D and D6D inhibitor. The ABMC-7 cell-based desaturase assay suggests that CP-24879 could decrease the generation of leukotriene C4 and AA via nonspecifically inhibiting D5D and D6D activity [96]. However, the inhibition of D6D may raise safety concerns, since inadequate D6D is correlating with chronic inflammatory diseases, such as diabetic neuropathy and atopic eczema [97,98]. In contrast, sesamin and curcumin have better specificity on D5D. In liver microsome assay, curcumin could inhibit 49% of D5D and 18% D6D, whereas sesamin barely inhibits D6D, D9D, and D12D [99,100]. The anti-cancer properties of sesamin and curcumin have been widely studied in different types of cancers [101,102]. Notably, curcumin has been involved in several clinical trials (stage I to III) for the treatment of breast, prostate, and cervical and uterine cancers [101]. Although many studies have demonstrated the effect of sesamin and curcumin on cancer *in vivo* and *in vitro* [103], [104], [105], [106], [107]. We are still unclear what is the role of D5D in the anti-cancer properties of sesamin and curcumin. Given that inflammatory cytokines, such as TNF-α and NF-κB, are important downstream targets of sesamin and curcumin [108,109], these natural inhibitors may affect inflammatory tumor microenvironment (TME) via regulating D5D activity. Furthermore, several selective D5D inhibitors have been identified by Takeda Pharmaceutical Company, such as D5D-IN-326, T-3364366, and 3,5-diphenyl-4-methyl-1,3-oxazolidin-2-ones; however, the effect and mechanism of these molecules have only been evaluated in metabolic diseases (such as insulin resistance, obesity, and atherosclerosis) rather than cancer [110], [111], [112], [113]. Additionally, another molecule iminodibenzyl has recently been found as a promising D5D inhibitor. Iminodibenzyl could redirect COX-2 catalyzed DGLA peroxidation, resulting in the generation of 8-HOA in cancer cells [114,115]. The slow advance of the development of D5D inhibitors may be reasoned by (1) the missing identification of the fine crystal structure of the D5D enzyme and (2) the difficulty to obtain D5D with high stability and activity [10]. Another obstacle is the current methods to quantify PUFAs metabolites of D5D are laborious and time-consuming. Thus, a faster and reliable method needs to be established for accelerating the development of D5D inhibitors for cancer treatment. The high sensitive electrochemical sensor may be the answer. For instance, a recent study has demonstrated that a 2D nanomaterial Ti3C2 MXene-based sensor could simplify the procedures for assessing PGE_2_ and 8-HOA concentration in cell lysis, tumor tissues, and blood [116].

## Discussion and conclusion

PUFAs are essential nutrition in our daily diet [117]. Unbalanced PUFAs uptake is associated with various diseases statuses, including inflammation, cardiovascular diseases, type 2 diabetes, and cancer [9,32,45]. According to the traditional concept, the n-3 PUFAs are anti-inflammatory, whereas n-6 PUFAs are pro-inflammatory [118]. Indeed, AA could be produced in the n-6 PUFAs pathway, resulting in the production of PGs by COX-2 [117,118]. PGs, especially PGE_2_, play a critical role in immune diseases and inflammation through regulating mast cell activation, T_h_1 cell differentiation, T_h_17 cell proliferation, and cytokine production [119], [120], [121]. Give that inflammation is a key factor in cancer progression, previous studies have investigated the role of the n-6 PUFAs pathway in cancers [77,[122], [123], [124], [125]]. The COX-2/PGE_2_ axis could directly activate EP receptors on cancer cells, resulting in cancer cell proliferation, invasion, EMT, and angiogenesis [52,54,79,126,127]. Additionally, PGE_2_ from cancer cells also could affect EP receptors on endothelial cells in a paracrine pattern [128], [129], [130], [131]. Moreover, EP receptors are presenting in immune cells, such as MDSCs, macrophage, NK cells, dendritic cells, and T lymphocytes [53,[132], [133], [134], [135], [136], [137]]. The cancer cell or immune cell-derived PGE_2_ could bind to these EP receptors, creating an immune suppressor TME [47]. Therefore, suppression of PGE_2_ production in cancer cells by using COX-2 inhibitors may be a promising anti-cancer strategy.

However, clinical trials suggest that COX-2 inhibitors fail to improve the overall survival and progression-free survival of cancer patients [67,138]. Even worse, the long-term and high-dose COX-2 inhibitors for cancer patients may cause severe side effects, such as myocardial infarction [64,66,139]. Therefore, it is essential to develop a better strategy for cancer therapy by regulating the AA/PGE_2_ axis. Recent studies have revealed that D5D could be a desirable target for cancer treatment [14,81,82]. D5D is the rate-limiting enzyme catalyzing the formation of AA from DGLA [9]. Inhibition of D5D, either protein expression or activity, suppresses the production of AA and PGE_2_ [84], [85], [86]. Interestingly, in the cellular condition of D5D inhibition, DGLA could be converted to a distinct free radical byproduct, 8-HOA by COX-2 in cancer cells. 8-HOA inhibits cancer cell proliferation, migration, invasion, and promotes apoptosis via decreasing HDAC activity and YAP1/TAZ pathway in cancer cells. Consequently, cancer growth could be inhibited by the decrease of PGE_2_ and the increase of 8-HOA levels [82,[84], [85], [86],^88^,^89^]. The benefit of RNAi-based genetical knockdown of D5D, such as siRNA, shRNA, and RNA nanoparticles, have been confirmed in colon, pancreatic, breast, and lung cancers *in vitro* and *in vivo* [6,7,[83], [84], [85], [86],^89^]. Additionally, small molecule D5D inhibitors also have been identified in cell-based or rat liver microsomes assays, including sesamin, curcumin, D5D-IN-326, CP-24879, iminodibenzyl, etc [94,95,110,112,115,140]. Although iminodibenzyl efficiently suppresses lung cancer cell growth and metastasis [115], more studies need to be done to further explore the effect and mechanisms of other D5D small molecule inhibitors in cancer. Moreover, D5D may have different roles in cancer by simultaneously regulating multiple pathways. For example, a recent study indicates that D5D expression is differential in mesenchymal-type and intestinal-type gastric cancer cells. D5D may serve as an essential enzyme in cancer cell ferroptosis [5]. Therefore, it is plausible that inhibition of D5D may activate apoptosis but suppress ferroptosis in cancer cells. Elucidating why D5D has the opposite effect on apoptosis versus ferroptosis in cancer cells may help us to develop a better anti-cancer strategy in the future.

In conclusion, D5D is the key enzyme for regulating PUFA synthesis. The D5D and certain SNPs expressions are associated with cancer progression. In the n-6 PUFA synthesis pathway, DGLA is catalyzed by D5D to form AA, which is a precursor of PGE_2_, an inflammation mediator. The COX-2 catalyzed PGE_2_ could activate EP receptors and promote cancer cell growth and metastasis via regulating tumor microenvironment and immunosuppression. While the opposite function of D5D has been revealed in recent studies that D5D could trigger ferroptosis response in cancer cells via manipulating PUFA synthesis, leading to programmed cancer cell death. Additionally, in case of the absence of D5D, the downstream enzyme COX-2 also could directly catalyze DGLA peroxidation, resulting in the generation of a distinct anti-cancer free radical byproduct, 8-HOA in cancer cells. It has been demonstrated that inhibition of D5D could activate the apoptosis pathway but suppress cancer cell survival, proliferation, migration, and invasion. All these reports have suggested that D5D may serve as the central hub and ideal target for regulating different aspects of cancer progression. Despite the high efficiency of RNA nanoparticle-based siRNA treatment, continued research into selective D5D inhibitors may help elucidate the controversial mechanisms underlying PUFAs metabolism in cancer and may aid the development of novel therapeutical strategies.

## CRediT authorship contribution statement

**Lizhi Pang:** Conceptualization, Visualization, Writing – original draft. **Harshit Shah:** Writing – review & editing. **Yi Xu:** Writing – review & editing. **Steven Qian:** Conceptualization, Supervision, Writing – original draft.

## Declaration of Competing Interest

Steven Qian is an inventor on a patent (US-2019070193-A1) related to this work filed by NDSU Research Foundation. The authors declare no other competing interests.
